# Continuous Delivery of Blockchain Distributed Applications

**DOI:** 10.3390/s22010128

**Published:** 2021-12-25

**Authors:** Tomasz Górski

**Affiliations:** Department of Computer Science, Polish Naval Academy of the Heroes of Westerplatte (PNA), Śmidowicza 69, 81-127 Gdynia, Poland; t.gorski@amw.gdynia.pl

**Keywords:** blockchain, continuous delivery, 1+5 architectural views model, model-driven development

## Abstract

Ensuring a production-ready state of the application under development is the imminent feature of the Continuous Delivery (CD) approach. In a blockchain network, nodes communicate and store data in a distributed manner. Each node executes the same business application but operates in a distinct execution environment. The literature lacks research focusing on continuous practices for blockchain and Distributed Ledger Technology (DLT). Specifically, it lacks such works with support for both design and deployment. The author has proposed a solution that takes into account the continuous delivery of a business application to diverse deployment environments in the DLT network. As a result, two continuous delivery pipelines have been implemented using the Jenkins automation server. The first pipeline prepares a business application whereas the second one generates complete node deployment packages. As a result, the framework ensures the deployment package in the actual version of the business application with the node-specific up-to-date version of deployment configuration files. The *Smart Contract Design Pattern* has been used when building a business application. The modeling aspect of blockchain network installation has required using Unified Modeling Language (UML) and the *UML Profile for Distributed Ledger Deployment*. The refined model-to-code transformation generates deployment configurations for nodes. Both the business application and deployment configurations are stored in the GitHub repositories. For the sake of verification, tests have been conducted for the electricity consumption and supply management system designed for prosumers of renewable energy.

## 1. Introduction

**Figure 1:**
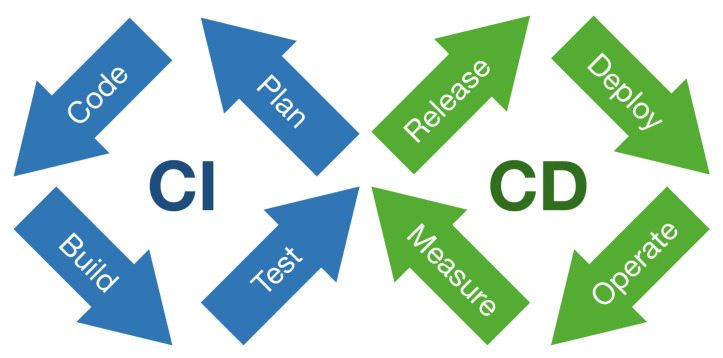
The Möbius strip in continuous software engineering.

The fundamental principle of the Agile Manifesto underlines the importance of early and continuous delivery of software that meets the needs of the customer [1]. The author has distinguished, and provided abbreviations for, the following notions: continuous integration (CI), continuous delivery (CD), and continuous deployment (CDT). CI practice involves that software is integrated continuously during development. The practice encompasses automation of software builds and testing. The basis for a CI is a version control environment. The most popular is GitHub. The service ensures the source code version control in a distributed manner using Git. The Git distributed version control has its own branching model [2]. Generally, developers frequently merge their code with release or main branches [3]. The new or changed code is incorporated into a build and checked by automated tests. Test automation ensures checking that the application works correctly in case a new commit is merged into the release branch. The practice raises team productivity by frequent releases. Iterative automated testing elevates software quality in the CI approach. The CD approach goes even further in software development automation. It aims to enable on-demand software release. The practice employs a set of stages including the acceptance tests and release process. Both those stages are automated. The release process and automated acceptance tests allow for application under development of on-demand deployment. Humble and Farley [4] present the comprehensive description of the continuous delivery process. They have defined the notion of the deployment pipeline as an automated process of tasks, which is responsible for producing release. CD practice requires specific governance to act properly, i.e., infrastructure, data, and configuration management. The CDT approach elevates automation on even higher level. Each release is deployed automatically onto the user acceptance test environment or even production one. CDT is a push-based practice. On the contrary, CD approach is a pull-based one. Figure 1 presents loop of steps in continuous integration and delivery approaches.

The loop describes all steps of the DevOps approach. Recently, the IEEE Standard for DevOps has been approved and published (IEEE Std 2675-2021) [5]. The standard provides requirements and guidance on the implementation of DevOps to define, control, and improve software life cycle processes. It applies within an organization or a project for building and deploying software reliably.

Table 1 shows steps from the DevOps loop supported by each presented practice.

The author has observed increasing interest in continuous approaches. Shahin et al. [6] presented analysis of continuous practices. They have concentrated on both methodological and practical aspects of implementing those practices. They have found the following important topics: reducing build and test time, automation of tests, raising the scalability of deployment pipelines, and elevating of the deployment process reliability. They have also enumerated essential elements in implementing continuous approaches and underlined suitable infrastructure, testing, highly skilled programmers, and proven design patterns.

**Figure 2:**
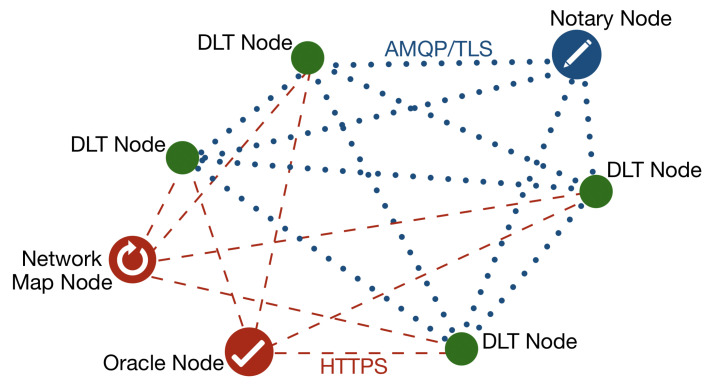
Types of nodes in the R3 Corda network.

Xu et al. [7] provide the following definition of a distributed ledger: “A distributed ledger is an append-only store of transactions which is distributed across many machines”. Details of transactions between nodes are stored in at least two of them. That store has a form of chain of interconnected transactions. We can add a new transaction. But we can not modify of delete existing one. The following transaction may modify a previous one, but both of them are stored in a chain. Each transaction is created in a decentralized manner with using a consensus algorithm that guarantees data integrity. A consensus algorithm is a fundamental component of distributed ledger and blockchain that ensures synchronization among multiple peers. The author draws attention to two consensus algorithms: proof-of-work (PoW), and proof-of-stake (PoS). Using PoW consensus algorithm involves solving a cryptographic puzzle to generate a hash [8]. That computationally intensive algorithm uses a lot of electricity and takes time. Thus, the PoS algorithm has been proposed that reaches the consensus by proving the stake ownership [9]. The author would also point out the fact that there are works on alternative approaches [10,11]. There are two major distributed ledger kinds. In a public ledger, also known as the permissionless ledger, participants using transactions may change the state of the ledger. It is important that the stored information is transparent. In such a type of ledger, privacy may be compromised. On the other hand, in a private ledger only authorized participants may take part in transactions. So, the privacy of transactions is ensured. A permissioned ledger is another term to name a private ledger. Chowdhury et al. [12] have compared a set of distributed ledger platforms. Among others, they have chosen Hyperledger Fabric, Ethereum, IOTA, Multichain, and R3 Corda. The R3 Corda is a private ledger where consensus involves only nodes that participate in the transaction. The fact has a vast impact on scalability. Transactions occur between DLT nodes and there are signed by the Notary node. There are also a Network Map node and Oracle nodes in the ledger. In a transaction take part two DLT nodes and one Notary node. The R3 Corda’s block creation time is 0.5–2.0 [s], which places the framework among the fastest. Besides, the usage of energy by the framework is almost negligible. A DLT node hosts distributed applications and services. The framework operates using services, the main ones being: oracle, notary, network map, and permissioning. A Corda network is a fully connected graph. The communication among DLT nodes and the Notary node is done via Advanced Message Queuing Protocol over Transport Layer Security (AMQP/TLS). Moreover, it uses Hypertext Transfer Protocol Secure (HTTPS) for the communication of DLT nodes with the Network Map and Oracle nodes. Figure 2 presents types of nodes in the R3 Corda distributed ledger network.

The solution presented in this paper constitutes a delivery component of a wider continuous deployment approach outlined by Górski in [13]. The contribution is the *BinCD*, a continuous delivery framework for blockchain. Distributed ledger introduces an additional level of difficulty for continuous delivery. In addition to ensuring the proper functioning of the business application, we are dealing with a variety of deployment configurations for the distributed ledger network nodes. Each of the business-relevant nodes (DLT nodes in case of R3 Corda) in the ledger may have a completely different specification of the deployment configuration. The *BinCD* approach encompasses the modeling support for the *Deployment* view in form of the *UML Profile for Distributed Ledger Deployment*. Górski in [14] has described the refined profile that encompasses node deployment configuration for R3 Corda versions from 4.3 to 4.6. Additionally, the *UML2Deployment* transformation has been refactored. It automates the deployment of various runtime environments for blockchain networks [15]. The framework also includes source code management and version control for generated deployment scripts and the smart contract Java application. To achieve this, the GitHub distributed version control environment has been used. The approach encompasses checking the consistency between generated deployment scripts and the UML Deployment model. Besides, deployment pipelines have been designed that automate the release process for the distributed application and node deployment configuration scripts. The Jenkins open source automation servers have been used. For verifying smart contracts, both unit and integration tests have been included in the Java application deployment pipeline.

The paper is arranged as follows. Section 2 discusses the related work. Section 3 presents the design of the *BinCD* solution that provides up-to-date deployment scripts and smart contracts application (Java) for DLT nodes configuration. The section encompasses a description of continuous delivery pipelines designed in the Jenkins open source automation server. Moreover, in that section, it have been also presented design of refined *UML2Deployment* transformation. Section 4 introduces the method of validation of the continuous delivery solution that allows for checking the consistency between generated deployment scripts and source models. The section also outlines tests of the distributed application. Section 5 presents discussion. Section 6 concludes the paper and shows directions for further work.

## 2. Related Work

This work focuses on the Continuous Delivery practice, blockchain technology, UML, and the Model-Driven Development (MDD) methodology. Therefore, the search has considered articles on these four topics and divided the results of the literature review into corresponding paragraphs. The first one presents papers that discuss recent developments in continuous practices. The second paragraph discusses articles which show the recent developments in blockchain technology. Special attention has been paid to the matter of blockchain solutions for the renewable energy sector. The next paragraph encloses studies that show the newest applications of UML. The last one focuses on the research advances in employing MDD. Emphasis has been put on finding such uses in blockchain solutions.

There are approaches and best practices of continuous delivery described in the literature [4]. Nevertheless, the implementation can be difficult in practice. Laukkanen et al. [16] have selected papers considering continuous delivery adoption problems, causes, and solutions. Integration and testing problems have been the most often reported ones. System design and testing problems have been revealed as the most critical ones. Particularly, testing in continuous integration is proliferating in the literature. Prado Lima et al. [17] present papers devoted to prioritization of test cases in continuous integration. In that field, Yang et al. [18] show that a reward function based on historical data can elevate the efficiency of such prioritization. Moreover, Yu et al. [19] reviewed papers that show the using of a continuous integration environment for non-functional requirements testing. Abdalkareem et al. [20] have shortened the execution time of the continuous integration process by identifying commits that can be omitted. The proposed prototype tool works with Git repositories. Debroy and Miller [21] showed actions to overcome challenges in implementing continuous practices. They used custom images for building agents to handle microservice dependencies. They introduced orchestration to manage resources in order to keep infrastructure costs low. Finally, keeping build and release times short required employing an orchestrator, such as Kubernetes, to handle scaling. Gallaba et al. [22] have proposed a tool to analyze feature misuse of Travis CI. The same environment, Travis CI, has been used by Saidani et al. [23] to analyze refactoring practices in continuous integration. In the work, the Jenkins open-source automation server has been used, which is widely used by the community and researchers [24,25]. Recently, Leite et al. [26] have examined continuous delivery practice. They have analyzed the structure of DevOps teams and communication between them. So the work seems to be timely.

Blockchain is one of the most disruptive technologies. Both papers, by Monrat et al. [27], and Al-Jaroodi et al. [28] examine the benefits and difficulties of using the technology for business applications. The work has only focused on a few of them. Blockchain is widely used for the management of healthcare data [29]. An example of the Electronic Health Record system that applies blockchain has been presented by Shahnaz et al. [30]. Blockchain finds a lot of uses in the energy sector. Especially, distributed energy systems of prosumers employ the technology to manage the flow of electricity. Saxena et al. [31] have developed a blockchain-based residential energy trading system that takes into account the preferences of prosumers so as to reduce the demand for energy. They have used permissioned Hyperledger Fabric [32]. Jamil et al. [33] have introduced a predictive energy trading system that helps in scheduling energy generation from renewable sources. Researchers and practitioners use various blockchain frameworks. An extensive comparison of permissioned and permissionless blockchain frameworks have been done by Chowdhury et al. [12].

Practitioners use Unified Modeling Language for software architecture modeling. They depict architecture from various viewpoints. Ozkaya et al. [34] surveyed 109 practitioners with diverse profiles to discover usage of UML diagrams and architectural viewpoints. It turned out that information (99%) and functional (96%) viewpoints are the most proliferated. We see the need to put more emphasis on the deployment viewpoint. They have also surveyed professionals on the usage of UML diagrams in modeling different aspects of software architecture. The study shows that the UML class diagram is commonly used for data structure modeling (85%) and UML deployment diagram is applied to physical structure modeling (71%) and functional to physical components mapping (53%). It is worth mentioning that the UML activity diagram finds its application for software build and release processes modeling (20–22%).

Table 2 contains the results of UML diagrams usage, where the numbers mean the percentage of professionals who selected the considered diagram to model a chosen aspect of software architecture.

Additionally, the uses of UML are still the focus of research. For example, Chavez et al. [35] have worked on ensuring cohesion between the Java source code and the UML class diagram. Software models that are expressed in UML may use Object Constraint Language (OCL) to provide precise semantics. Lu et al. [36] show the application of OCL constraints to medical rules in cancer registries. They have also used UML class diagrams. New research shows more and more uses of UML combined with MDD. Assunção et al. [37] have used UML class diagrams to generate Product Line Architecture variants. Arora et al. [38] have applied a bio-inspired methodology on concurrent sub-part of a UML activity diagram to obtain various feasible test scenarios. Arcaini et al. [39] present the technique that merges tests designed for subsystems to obtain tests for the whole system model. Abouzahra et al. [40] have done a literature review in the context of MDD model composition. One of the findings is a lack of backward compatibility in proposed approaches. Moradi et al. [41] have shown a solution that transforms the model of services into executable web services. We have also found works that deal with the efficiency of transformations. Basciani et al. [42] show reusability capabilities of existing transformations by chaining them for designing new ones. Besides, Panach et al. [43] report that the quality of the software developed following MDD is significantly better than the code written manually only for complex problems.

The new field of application of model-driven development is also blockchain technology. One of the key elements in a blockchain is a smart contract. Zou et al. [44] conducted an analysis to discover the actual obstacles that developers have to overcome while developing smart contracts. Results revealed that the source code of smart contracts is compromised as far as security is concerned. Besides, existing frameworks are rudimentary and there are many limitations in programming languages. Górski [14] has shown the flexible manner for designing smart contracts in a permissioned distributed ledger. He has proposed the *Smart Contract Design Pattern*. As far as MDD is concerned, Xu et al. [7] outline two transformations applied to blockchain technology. The first one uses cooperating business processes and generates a smart contract for them. The second transformation generates blockchain registries for commodities, digital assets, and ownership titles. They have used the Ethereum blockchain. Górski and Bednarski [15] describe the model-to-code transformation, which creates deployment configuration files for blockchain nodes by using the UML Deployment model. The model employs extensibility mechanisms from *UML Profile for Distributed Ledger Deployment* [14]. Moreover, Gao et al. [45] propose a tool prototype to automatically detect bugs and validate smart contracts.

It is worth emphasizing that Laukkanen et al. [16] in their survey pointed out that the system design and build design topics had the least reported solutions in the Continuous Delivery domain. Support for modeling has been incorporated into the *BinCD* solution. Smart contracts are the main focus of current research results on blockchain. These are the primary element of distributed applications. Besides, blockchain nodes constitute the deployment environment. The deployment environment, which is properly configured, hosts distributed applications. Ozkaya et al. [34] claim that the functional and information views are the most popular views in software architecture modeling. The paper has concentrated more on the deployment (*UML Profile for DLT Deployment*). But, it have been also included design assistance for smart contracts (*Smart Contract Design Pattern*). Continuous practices involve the automation of tasks in software development and IT operations. Thus, the paper pays attention to employing the MDD approach (*UML2Deployment* transformation) to the Deployment view of a blockchain system. The imminent element of continuous delivery is also version control. There have been used Git distributed version control and GitHub service. Zou et al. [44] claim that the development support of blockchain applications in existing tools is still incomplete. Thus, the Visual Paradigm modeling tool has been incorporated into the framework. Besides, the Jenkins automation server has been used for automated build release. The whole CD pipeline has been integrated, from the UML deployment model to the deployment package for each node in the distributed ledger network. Additionally, a parallel pipeline has been provided that builds Java distributed application and places it in every deployment package. As a result, a solution, which uses UML and MDD, has been obtained that provides deployment-ready packages for each distributed ledger node with the actual version of the Java distributed application and up-to-date deployment configuration.

## 3. The BinCD Framework Design

**Figure 3:**
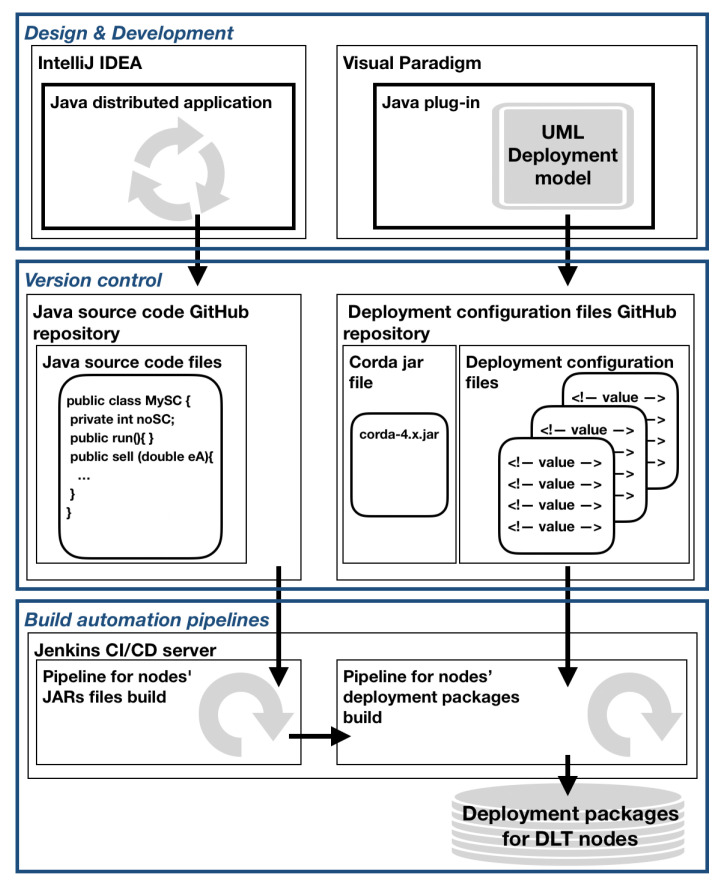
The BinCD framework overview.

The *BinCD* framework in its design employs various architectural principles. Firstly, there have been imposed *Modularity* and *Separation of responsibility* principles on the design of the *BinCD* approach. As a result, the following layers have been identified: Design & Development, Version control, and Build automation pipelines. Figure 3 depicts the overview of our *BinCD* framework to automate the generation of release packages with the distributed applications and blockchain node deployment configurations.

In the *Design & Development* layer, the *UML Deployment model* of the blockchain network has been placed. The model uses, as previously mentioned, the profile and the transformation plugin. The second module is a *Java distributed application* that realizes the *Sell energy* smart contract. The first module is designed in Visual Paradigm whereas the second one is developed in IntelliJ IDEA. GitHub repositories have been placed in the *Version control* layer. The first one encompasses the source code of the smart contract application and Corda’s actual version execution environment. The second repository consists of deployment configuration files for nodes. Both Jenkins pipelines have been put in the *Build automation pipelines* layer. The first one automates the generation of the smart contract application. The second pipeline automates the generation of the complete ZIP file that consists of the application, Coda runtime, and deployment configuration files for DLT nodes. Deployment packages for nodes are kept by the Jenkins server.

Next, elements of the *BinCD* framework have been presented. Firstly, the current version of the *UML Profile for Distributed Ledger Deployment* has been briefly outlined. Then the *UML2Deployment* transformation has been presented. The transformation has been adapted to store files in GitHub repositories. Lastly, delivery pipelines have been outlined that generate releasable deployment packages for distributed ledger nodes.

### 3.1. UML Profile for DLT Deployment

**Figure 4:**
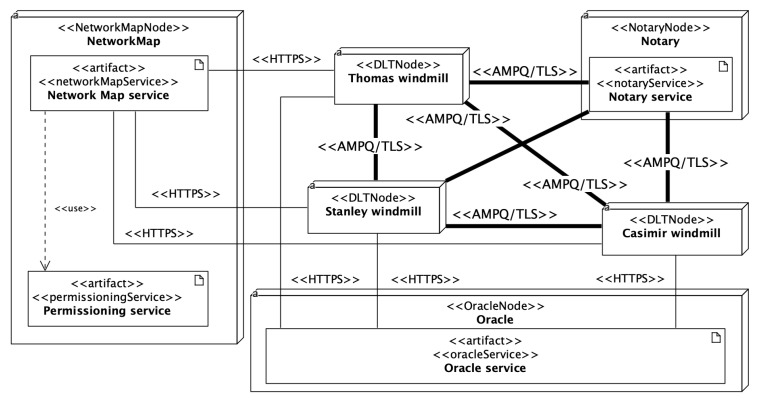
The test deployment environment in the UML Deployment diagram.

The author has previously thoroughly refined the profile [14]. In the design of the profile, he has applied the *Adjust the level of abstraction* architectural principle. The profile is defined at the Platform Specific Model (PSM) level to express the precise deployment configuration of the R3 Corda framework. But, it has been configured flexibly enough for modeling various versions of Corda (currently from 4.3 to 4.6). Unified Modeling Language offers constraints, tagged values, and stereotypes to extend its semantics [46]. Stereotypes have been employed to mark nodes, services, and communication protocols specific to the R3 Corda framework. The framework defines deployment parameters for each type of node. In the profile, the deployment parameter is represented by the tagged value. Tagged values are coupled with stereotypes of nodes. Stereotypes and tagged values, from the profile, have been used in the UML Deployment model of the ECSM system. Usually, various deployment environments are modeled, e.g., test, uat, staging, prod. UML package groups nodes and represents the deployment environment. Separate UML Deployment diagrams depict deployment environments. The profile can be found in the GitHub repository [47]. Figure 4 depicts the UML Deployment diagram of the test deployment environment defined for a part of the ECSM system.

### 3.2. UML2Deployment Transformation Design

**Figure 5:**
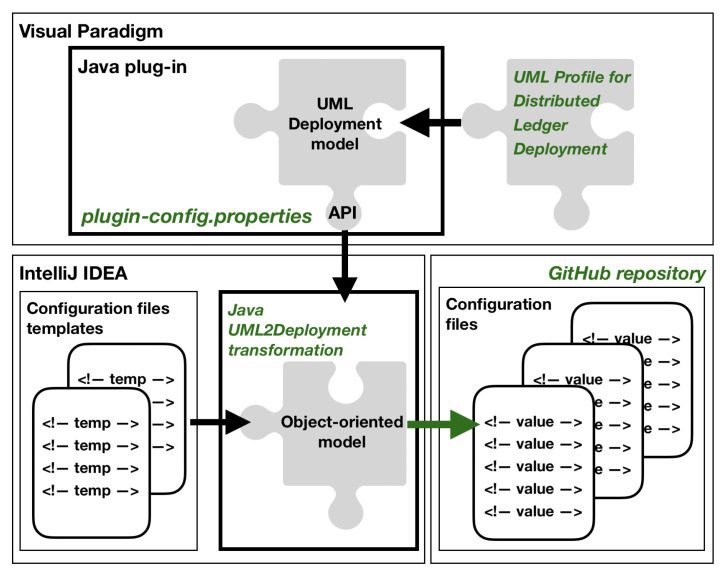
Overview of the transformation.

The continuous delivery framework incorporates model-to-code transformation for generating blockchain deployment scripts [15]. Figure 5 outlines the overview of the extended transformation (new or redesigned components are marked in green).

The source of the transformation is a UML Deployment model. The current version of the UML profile has been used. The second vital change in the design of the transformation is the ability to store generated deployment scripts at GitHub under Git version control. Having the *modularity* architectural principle in mind, the design of the transformation has been split into two main components: the UML Deployment model and the Object-oriented model. Following the *separation of responsibility* architectural principle, the transformation has been designed as two distinct Java applications. The main Java application generates configuration files using the content of a UML Deployment model. The application uses configuration files templates. The second application is designed as the Visual Paradigm plug-in that uses the transformation. The current version of the transformation incorporates the possibility of storing generated deployment scripts at GitHub service under Git distributed version control. The Visual Paradigm plugin has been configured to work in two modes: LOCAL and GIT. The first one was left for the sake of backward compatibility. When the plugin is set up for LOCAL mode, the application forces the user to select a local path to save generated files. With GIT mode turned on, generated deployment configuration files are automatically committed and pushed to the specified repository. The specification of work mode for the plugin is done with a dedicated property file, *plugin-config.properties*.

Table 3 contains options for configuring work modes for deployment environments.

**Figure 6:**
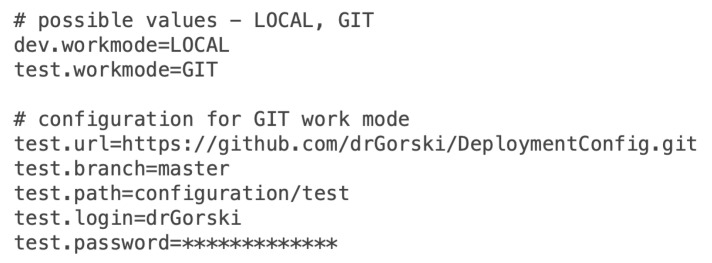
Example of the *plugin-config.properties* file.

An example of the *plugin-config.properties* file has been prepared that configures the transformation to work in GIT mode for *test* environment. The plugin reads the content of the *plugin-config.properties* file and sets the transformation to store generated files in *drGorski/DeploymentConfig* repository in *master* branch. The path is set to *configuration/test* value. Figure 6 shows the content of *plugin-config.properties* file.

The Java plugin reads the properties file and sets the destination for storing generated files. The plugin uses the *PluginConfiguration* class constructor to set the destination (in our example the *drGorski/DeploymentConfig/configuration/test* path). The Java plugin *NodeConfigGenerator* class invokes the *generate()* method of *CordaGenerator* class in the *UML2Deployment* transformation and passes generation destination. The transformation generates deployment files using *generateDeployNodesTasks()* method of *NodeGenerator* class and invokes *store()* method on destination. In the GIT mode configured, after executing the transformation for the selected environment, deployment configuration files are automatically sent to the repository. Examples of such generated deployment configuration files can be found in the *drGorski/DeploymentConfig* repository at the *configuration/test* path in *master* branch [48]. The source code of the redesigned transformation application and the Java plug-in with added integration with distributed version control is stored in GitHub repositories, [49,50] respectively. The transformation ensures consistency of the *UML Deployment model* with deployment configuration files of blockchain nodes.

### 3.3. Deployment Pipelines

At this stage, the source codes of the application and implementation configurations are managed in the version control system. The platform documentation describes the content of the node deployment package [51]:

- Corda run-time environment—*corda-4.6.jar*,
- All needed Cordapps JARs in /cordapps subdirectory:
  – *cordapp-ecsm-contracts-0.1.jar*,
  – *cordapp-ecsm-workflows-0.1.jar*,
- Node deployment configuration file—*node.config*.

**Figure 7:**
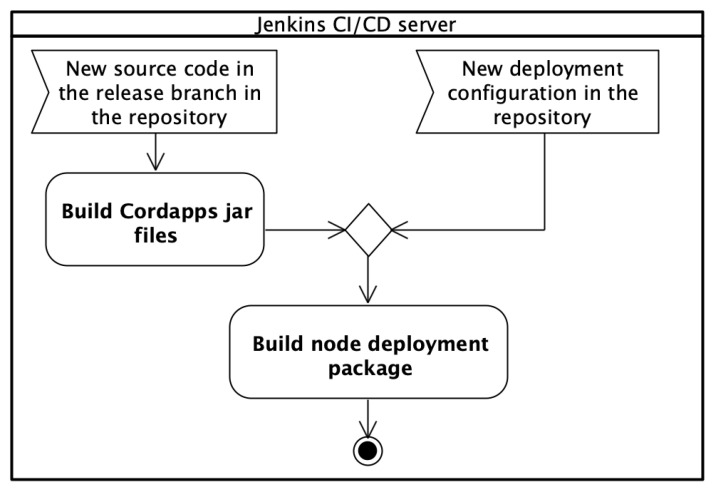
The BinCD solution deployment pipelines.

The complete deployment release for the entire DLT solution should be packed into a ZIP file. Concerning the example, this is the *cordaECSM.zip* file. Especially for more complex projects, it may be an even more elaborate deployment structure with multiple repositories. Thus, it is important to automate the preparation of deployment packages for nodes. The Jenkins automation server has been used for completing and storing node deployment packages. The main notion in the Jenkins automation server is a pipeline. A pipeline can be described as an automated process of generating a releasable package on the basis of software stored under version control. A pipeline defines your entire build process, which typically includes stages. A stage block defines a conceptually distinct subset of tasks. A single task tells Jenkins what to do at a particular step in the process. Proper node deployment package involves both business logic and deployment configuration details. Thus two Jenkins pipelines have been introduced (see Figure 7): *Build node deployment package* and *Build Cordapps jar files*.

The *Build node deployment package* pipeline is responsible for completing the full node deployment package on the basis of delivered Cordapps JAR files and deployment configuration ones. The *Build Cordapps jar files* pipeline has a helper role. This pipeline is responsible for building an up-to-date version of Cordapps JAR files. The pipeline is triggered when a new source code of business logic appears in the repository in the release branch. After finishing execution, the pipeline triggers the *Build node deployment package* pipeline. This split allows separating the Java source code of business logic from the node’s deployment configuration details. The main pipeline *Build node deployment package* is triggered automatically, always when a new deployment configuration is generated from the UML Deployment model. For test purposes, it is also possible to start this step manually by the user. At this stage, the *BinCD* solution determines for which nodes and environments packages should be built.

The *BinCD* has two paths of selection:

- When config files are added/changes it will build new packages only for the changed/added nodes,
- In case of changing the source code of Cordapps, new JAR files are built and all packages for nodes and environments are prepared with existing deployment configuration but new business logic.

Next, it has been shown the design of both pipelines.

#### 3.3.1. Build Node Deployment Package Pipeline

**Figure 8:**
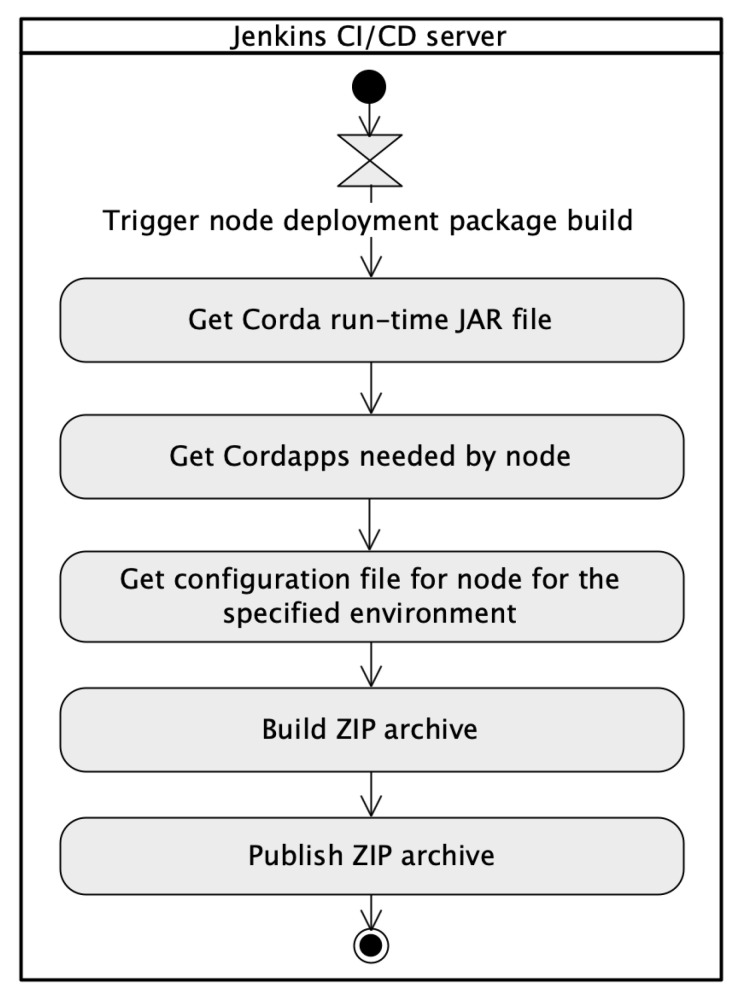
The *Build node deployment package* pipeline.

The stages in the *Build node deployment package* pipeline have been presented in the UML activity diagram (see Figure 8).

**Figure 9:**
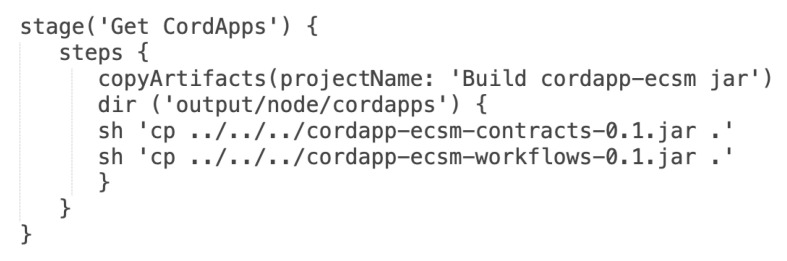
Groovy source code for *Get CordApps* pipeline stage.

There are two possible entry points to trigger execution of the *Build node deployment package* pipeline. The basic configuration of the pipeline invocation involves the use of a *cron* time-based job scheduler. It can be marked the ”Poll SCM” check-box and specify how often the Jenkins server has to monitor changes in the repository with deployment configuration [48]. Additionally, the pipeline has been configured to be started as a result of building another pipeline. The ”Build after other projects are built” check-box has been marked and set *Build cordapp-ecsm jar* pipeline to be watched. The intrinsic part of each node is the Corda platform JAR file. In the solution, the Corda run-time JAR file is stored in the same Git repository [48]. The first stage in the pipeline is the *Get Corda run-time JAR file*. The stage clones the current repository where the file is stored and copies it into a temporary directory which is used to build a ZIP archive. Moreover, the newest version of Cordapps is always used. Artifacts in their actual versions are copied to the directory for further use to set up the ZIP archive of the node. Figure 9 shows Groovy source code for the *Get Cordapps needed by node* pipeline stage. In the script, the abbreviated form has been used—the *Get CordApps*.

**Figure 10:**
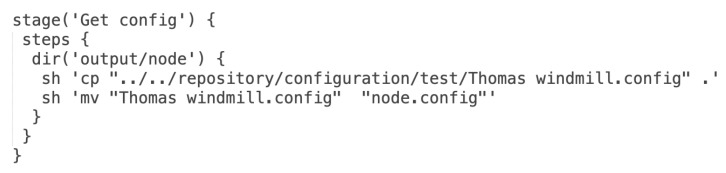
Groovy source code for the *Get config* stage in the pipeline.

When the configuration files are stored in the Git repository they can be used by the pipeline during the node package build process. The configuration files are copied to the proper directory for further use to create a ZIP archive. Figure 10 depicts Groovy source code for the *Get configuration file for node for the specified environment* pipeline stage. In the script, the abbreviated form has been used – the *Get config*.

**Figure 11:**
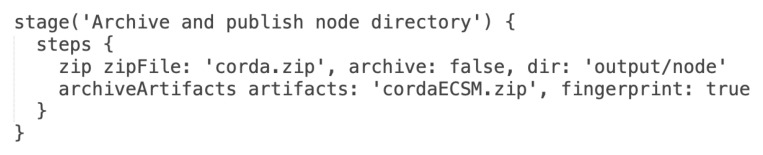
Groovy source code for *Archive and publish node directory* stages.

At this stage, all needed files are collected and located in proper directories and the final ZIP package is compressed and archived for usage during node deployment. Figure 11 presents Groovy source code for *Build ZIP archive* and *Publish ZIP archive* pipeline stages.

#### 3.3.2. Build Cordapps Jar Files Pipeline

The dedicated *Build Cordapps jar files* pipeline is the implementation of a continuous integration approach. In the R3 Corda framework, the business applications are called Cordapps. These are crucial elements and once built should be promoted across different environments and nodes without changes. The source code once tested should be reused in many places. Such an approach ensures that in all environments exactly the same version is delivered. The pipeline is responsible for building JAR files being deployed on the Corda platform. It is started each time when new source code appears in the repository and delivers deployment-ready JAR files in accordance with Corda platform requirements. Once built, the business application JAR files are used in various deployment environments. To achieve that, after finishing the execution of the *Build Cordapps jar files* pipeline, the *Build node deployment package* pipeline is triggered to build a complete node deployment package. That split allows for the separation of both topics: source code and deployment configuration. The *Build Cordapps jar files* pipeline has been also configured as a Groovy source code. The Groovy script clones the current version of the source code from the GitHub repository. Next, the script runs unit and integration tests and later calls the dedicated Gradle task responsible for preparing JAR files. Finally, the prepared files are archived and published in the Jenkins directory. Those files will be used by the *Build node deployment package* pipeline to set up the ZIP archive with the complete node deployment package.

**Figure 12:**
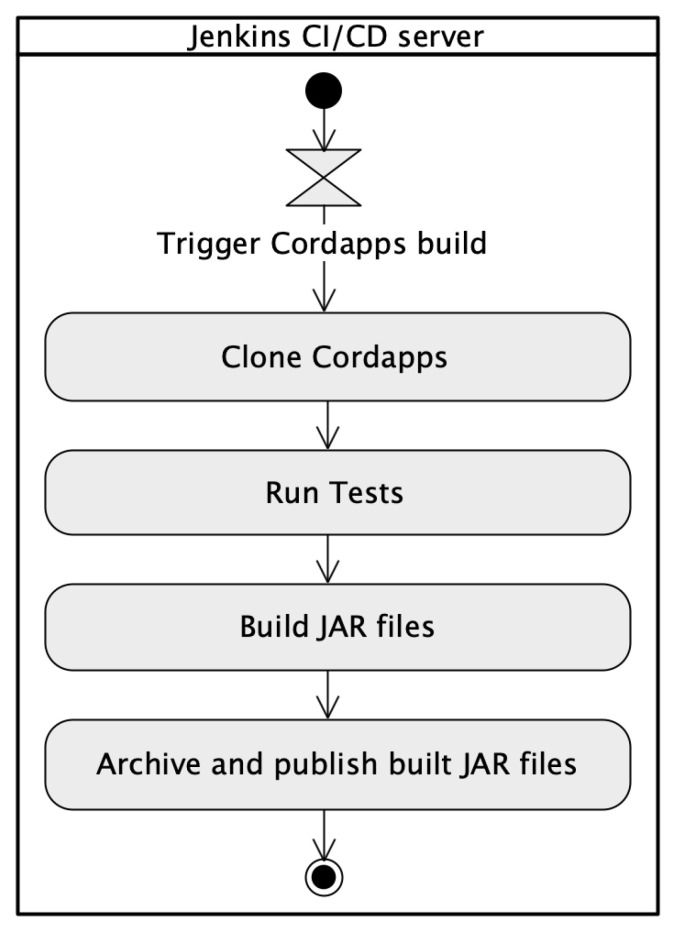
The UML activity diagram shows the *Build Cordapps jar files* pipeline.

Figure 12 shows the flow of events of the *Build Cordapps jar files* pipeline.

#### 3.3.3. Visualization of the Pipeline Execution

**Figure 13:**
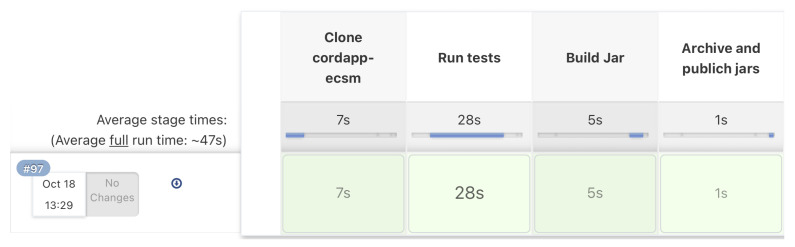
Results of the pipeline execution.

Each execution of the Groovy script for our configured pipelines can be visualized by the Jenkins server. The visualization of the pipeline execution allows monitoring of each stage and collecting statistics, e.g., *Average stage time*. At the end of both pipelines, there is an additional stage, the *Declarative: Post Actions*, technically closely tight with the selected automation server. After finalizing each of the pipelines, the workplace must be cleaned. All temporary files and directories that have been used during execution should be removed. Figure 13 shows metrics for the execution of the *Build Cordapps jar files* pipeline implemented in the Jenkins automation server. The pipeline execution time is under 50 s.

The Jenkins server with designed pipelines has been installed in the domain of the statutory project *model.amw.gdynia.pl*.

## 4. The Solution Validation

Validation determines whether a system or component satisfies requirements specified by the user [52]. In the work, validation is two-pronged. The first part means verifying the correctness of generated deployment configuration files. Secondly, it should be checked whether the business application works correctly. As far as deployment configurations are concerned, the consistency of deployment scripts with the UML Deployment model should be verified. A single deployment configuration file and the corresponding UML node are considered. The UML node comprises tagged values, t∈T. The script contains deployment configuration parameters, d∈D. The intersection of the two sets *D* and *T* is denoted by D∩T, and is the set containing all elements of *D* that also belong to *T* or similarly, all elements of *T* that also belong to *D*. It means checking that the intersection meets the following Equation (1).

(1) D∩T=D=T.

The cardinality of both sets should be the same. The deployment configuration parameter *d* is an ordered pair, $d=(n^{d},v^{d})$, where: $n^{d}$ is the name, and $v^{d}$ is the value of *d*. The tagged value *t* is an ordered pair, $t=(n^{t},v^{t})$, where: $n^{t}$ is the name, and $v^{t}$ is the value of *t*. For each d∈D must be t∈T with the same name and value (2).

(2) $$\underset{d\in D}{⋀}\underset{t\in T}{⋁}\left(n^{d}=n^{t}\right)\wedge \left(v^{d}=v^{t}\right).$$

Similarly, for each t∈T must be d∈D with the same name and value (3).

(3) $$\underset{t\in T}{⋀}\underset{d\in D}{⋁}\left(n^{t}=n^{d}\right)\wedge \left(v^{t}=v^{d}\right).$$

**Figure 14:**
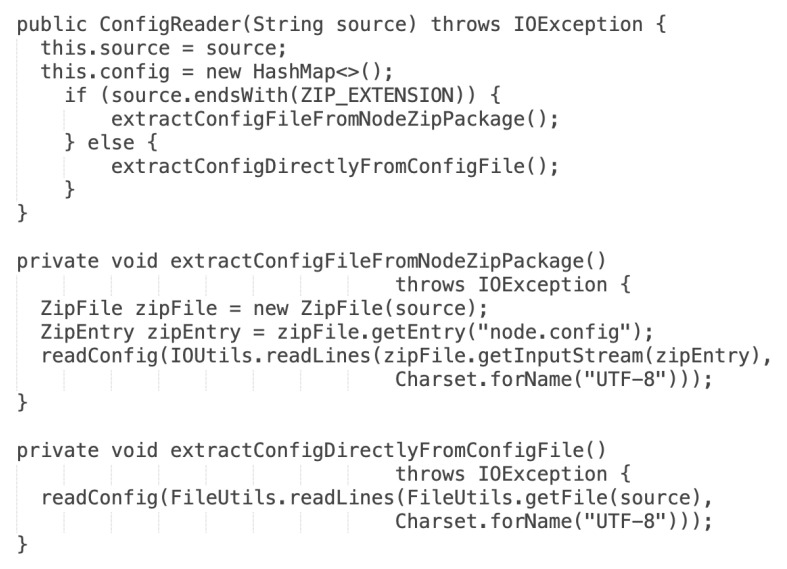
Source code with the selection of validation mode.

In the current version, the functionality of the verification has been expanded by checking the accordance of the ZIP package of deployment configuration files with the UML Deployment model. Figure 14 shows a source code that determines, which validation mode should be used.

**Figure 15:**
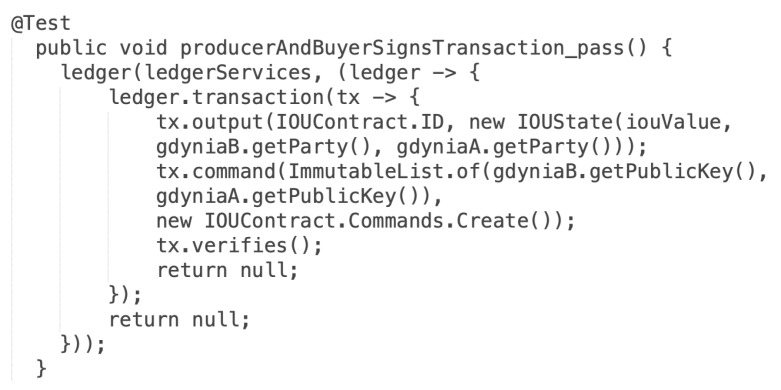
The source code of the positive test case.

The actual version recognizes what type of file was provided as input. In the case of providing the ZIP package, the validation application tries to find inside the archive the *node.config* file, and validation is run against the extracted file. When the input is recognized as a single configuration file, validation is run directly on the selected file. Ten test scenarios of the ECSM model have been designed to confirm the correctness of the transformation. Apart from verifying deployment configuration files, tests for the business application have been designed. The validation of the business application involves two kinds of tests: unit and integration. Unit tests are designed for individual methods in the business application, and integration tests verify the operation of the business application in blockchain nodes operating in the test environment, e.g., selling energy between two nodes. A dedicated the *IOUContractTest* class has been implemented for unit tests to verify rules in the smart contract. Figure 15 depicts the test case for one of the smart contract verification rules.

Integration tests go one step further and verify the end-to-end scenarios. The *DriverBasedTest* class has been implemented for integration tests. During an integration test, two blockchain nodes are configured and run. Next, the transaction is executed and committed. The test verifies whether the vaults of blockchain nodes store proper values of energy sold/bought. Both unit and integration tests have been prepared and run for the ECSM system [53]. As a result of tests, the *BinCD* approach proved to work properly. Both the model-to-code transformation and designed pipelines function as was intended. The redesigned validation application has been stored in the GitHub repository [54].

## 5. Discussion and Limitations

The author has noticed that in the field of Continuous Delivery, there is a limited amount of work done on the system design and build design [16]. Especially, distributed ledger and blockchain technologies are new spheres for the introduction of Continuous Delivery. Hence, modeling support has been incorporated into the *BinCD* solution for R3 Corda distributed ledger. Górski in [14] has focused on the Platform Specific Model to represent the exact deployment configuration of the R3 Corda framework. Such an approach lets the transformation rules be simpler and less prone to errors. On the other hand, the profile is configured in such an elastic fashion that it embraces versions of the Corda platform from 4.3 to 4.6. Therefore, in the transformation, file templates have been updated by adding new deployment parameters. The approach is open for handling the consecutive Corda platform versions. In the current work, version 4.6 of the Corda platform has been used. In course of further work, the solution will be upgraded to encompass versions up to 4.8.

Applying the simplicity architectural principle has been aimed at gaining linear order-of-growth of running time of our transformation. The complete running time of a program depends on two main metrics. These are the execution time of a statement and the frequency of its invocation. The first is the feature of the operating environment, but the latter is a property of the algorithm. As far as the frequency of executing each statement is concerned, the algorithm has been constructed to be a simple sequence of statements. One-dimensional dynamic collections like ArrayList as data structures have been used. As a consequence, it has been applied at most as a single *for* loop as a repetition control structure. Multi-dimensional data structures have been excluded to avoid nested repetition control structures. As a result, quadratic, cubic, or even exponential orders-of-growth have been eluded. In the current work, the number of tagged values in distributed ledger nodes doubled. So, the doubling hypothesis has also been verified. The running time of the *UML2Deployment* transformation still is under one second. Another factor is the number of DLT nodes. The performance analysis on bigger networks is still ahead. However, the estimation can be made. The distributed ledger network of the ECSM system, for testing purposes, has five nodes and generation takes approximately one second. So, for 1000 DLT nodes, it may take about 3 min. As far as memory usage is concerned, local reference variables to objects of classes from Java and Corda packages have been mainly used. So, the scope of visibility of those objects is very limited and quickly they become the interest of the garbage collector mechanism. The size of the Java collection to generate the deployment configuration of each node has also been estimated. The String object uses 40 bytes (object overhead, reference, hash, and padding) + (2c+24) bytes for char array, where *c* is the number of chars in the string. It was assumed that each tagged value is 10 chars long. Reference to String object uses another 8 bytes. Thus it was obtained estimation of 8+40+20+24=92 bytes per one tagged value. There are 107 tagged values in the DLT node so it is needed 9844 bytes for each node. It should be added another 24 bytes for the collection object itself. The result is 9868 bytes for the data structure for storing tagged values for a single node.

The runtime environment of the Corda platform *corda-4.6.jar* is needed to run Cordapps. The mentioned file can be downloaded from Corda’s official webpage, but the decision has been made to store it on project internal resources to avoid any performance or availability issues. The file should be hosted by a dedicated resource on which there is control. It has been decided to not increase the technological stack and selected *Git* repository as a place to store and host Corda platform JAR for the CD pipeline purposes. But, it should be remembered that *Git* is not optimized for the storage of large binary files. It would be worth considering using a dedicated tool like Artifactory to store and host such files. That may speed up the single pipeline execution.

## 6. Conclusions and Future Work

The paper introduces the continuous delivery approach for generating complete node deployment packages for a blockchain system. Moreover, the solution offers UML modeling support for the *Deployment* architectural view. The work uses *UML Profile for Distributed Ledger Deployment* at the Platform Specific Model for R3 Corda distributed ledger in version 4.6. The modeling support in the continuous delivery process has been included. The generation of complete deployment packages for the R3 Corda distributed ledger has been automated. Both smart contract application and deployment configuration files are placed in GitHub repositories under version control. The transformation application has been integrated with the Jenkins open source automation server, which uses deployment configuration files and the smart contract application stored in GitHub repositories. It is planned to expand the continuous delivery approach. Currently, the framework is able to generate releasable deployment packages. Additionally, it concentrates mainly on the *Deployment* view of the distributed ledger design. It is planned to enhance the solution by enabling the selection of the blockchain platform. The considered platform for inclusion in the approach is the HyperLedger Fabric. More attention is going to be put to the source code and test generation for smart contracts.To achieve that, the modeling support for the business application should also be added. This work moves toward the complete solution that will combine elements from all perspectives of the 1+5 architectural views model.

## Figures and Tables

**Table 1:** The DevOps loop steps supported by each presented practice.

DevOps Loop Step	CI	CD	CDT
Plan	*√*	*√*	*√*
Code	*√*	*√*	*√*
Build	*√*	*√*	*√*
Test	*√*	*√*	*√*
Release	–	*√*	*√*
Deploy	–	Manually	Automatically
Operate	–	–	–
Measure	–	–	–

**Table 2:** Usage of UML diagrams by professionals.

UML Diagram	Software Architecture Modeling
class	data structure (85%), functional structure (71%)
deployment	physical structure (71%), functional to physical components mapping (53%)
activity	data flow (65%), software build and release processes (20–22%)
sequence	data lifecycle models (47%)
component	software module structure (47%), system configuration (21%)
package	software module structure (47%)

**Table 3:** Properties for configuration of the transformation work modes.

Property	Description
workmode	Sets up work mode for the environment.
url	GIT repository URL for work mode.
branch	Branch name to store config files.
path	Branch path to store config files.
login	GIT credentials—login.
password	GIT credentials—password.

## Data Availability

Not applicable.
